# Hemispheric Lateralization of Visuospatial Attention Is Independent of Language Production on Right-Handers: Evidence From Functional Near-Infrared Spectroscopy

**DOI:** 10.3389/fneur.2021.784821

**Published:** 2022-01-14

**Authors:** Gaoding Jia, Guangfang Liu, Haijing Niu

**Affiliations:** State Key Laboratory of Cognitive Neuroscience and Learning & IDG/McGovern Institute for Brain Research, Beijing Normal University, Beijing, China

**Keywords:** interaction, functional lateralization, language production, visuospatial attention, handedness, fNIRS

## Abstract

It is well-established that visuospatial attention is mainly lateralized to the right hemisphere, whereas language production is mainly left-lateralized. However, there is a significant controversy regarding how these two kinds of lateralization interact with each other. The present research used functional near-infrared spectroscopy (fNIRS) to examine whether visuospatial attention is indeed right-lateralized, whereas language production is left-lateralized, and more importantly, whether the extent of lateralization in the visuospatial task is correlated with that in the task involving language. Specifically, fifty-two healthy right-handed participants participated in this study. Multiple-channel fNIRS technique was utilized to record the cerebral hemodynamic changes when participants were engaged in naming objects depicted in pictures (the picture naming task) or judging whether a presented line was bisected correctly (the landmark task). The degree of hemispheric lateralization was quantified according to the activation difference between the left and right hemispheres. We found that the picture-naming task predominantly activated the inferior frontal gyrus (IFG) of the left hemisphere. In contrast, the landmark task predominantly activated the inferior parietal sulcus (IPS) and superior parietal lobule (SPL) of the right hemisphere. The quantitative calculation of the laterality index also showed a left-lateralized distribution for the picture-naming task and a right-lateralized distribution for the landmark task. Intriguingly, the correlation analysis revealed no significant correlation between the laterality indices of these two tasks. Our findings support the independent hypothesis, suggesting that different cognitive tasks may engender lateralized processing in the brain, but these lateralized activities may be independent of each other. Meanwhile, we stress the importance of handedness in understanding the relationship between functional asymmetries. Methodologically, we demonstrated the effectiveness of using the multichannel fNIRS technique to investigate the hemispheric specialization of different cognitive tasks and their lateralization relations between different tasks. Our findings and methods may have important implications for future research to explore lateralization-related issues in individuals with neural pathologies.

## Introduction

Functional cerebral lateralization refers to functional differences between homologous regions of the left and right hemispheres. It is associated with information-processing functions (1, 2), and reflects human brain development (3, 4). Functional lateralization has been found using various approaches, such as split-brain patients (5), sodium amytal injection (6), and non-invasive neuroimaging (7–14). Those studies reveal that the left hemisphere is dominant in language-related processing, such as writing (12), speech perception (3), and language production (4), and that the right hemisphere is dominant in various non-verbal abilities, such as facial recognition (13), emotion expression (14), and visuospatial perception (11). Accordingly, it seems that the hemispheres complement each other in the intact brain. For instance, language processing is parallel in the two hemispheres, with the left hemisphere responsible for phonological processing and the right hemisphere responsible for prosodic processing (15). Such complementary hemispheric specialization in information processing may indicate a mechanism underlying the ontogenesis of functional lateralization. Thus, a question arises whether being left-lateralized for one function implies being right-lateralized for another. If so, a correlation between these functional cerebral asymmetries should be observed. However, the relationship of functional asymmetries has not been understood well-because only a few studies assess the relationship in the same sample.

Studies investigating the relationship between functional asymmetries in humans usually involve language production and visuospatial attention (7, 16–21), two essential cerebral functions mainly lateralized in the left hemisphere (22) and right hemisphere (11), respectively. Two opposite hypotheses have been examined (23, 24). The crowding hypothesis supported a “causal” relationship of the division of functions between hemispheres, suggesting that the lateralization of one function would cause the opposite lateralization of the other (25, 26). Evidence from fMRI imaging on left-handed participants (7, 18) found that the magnitude of lateralization for language production was negatively correlated with that of spatial attention, implying a mirrored brain organization of these two lateralized functions in these populations. For example, a significant slope of −8.7 was observed in a linear regression model where the laterality indices from line bisection judgment task (i.e., landmark task) and sentence production were served as independent and dependent variables with 99 stronger left-handers (18). According to the statistical hypothesis (17, 19), on the other hand, different functions lateralize independently (17, 19), suggesting that lateralization of language-dependent processing and that of spatial processing are independent of each other (17, 20, 27). For example, in a study of fMRI imaging on a group of 48 left- and 107 right-handed participants, researchers only revealed a weak correlation (i.e., *r* = −*0.18*) between lateralization indices derived from word generation and landmark tasks (19). Evidence from the resting-state also reported that the laterality index of language-related regions could not predict that of spatial attention-related regions in right-handed participants (*r* = *0.07, n* = *62*) (28).

Accordingly, the inconsistency regarding the relationship between language production and spatial attention seems to lie in the hand preference of the participants. The relationship between handedness and language lateralization has been well-documented (29, 30): it is not a mirror correlation (i.e., handedness is opposite from the language-lateralized hemisphere) (31), but it maintains a robust correlation (32, 33). For instance, children with language and manual preference dominant in the same hemisphere outperformed those with opposite hemispheres dominant for each function in reading performance (34). As a result, if language production is related to spatial attention in some causal way among left-handers, the “causal” relationship should be observed on right-handers as well. Presumably, right-handers will show a stronger association between these functional asymmetries, given their overall lower phenotypic variability and stronger population-level lateralization biases than left-handers (30, 35). Thus, the effect of handedness on the relationship between functional asymmetries lacks further evidence.

Methodologically, functional near-infrared spectroscopy (fNIRS) is a non-invasive brain imaging technique that measures the changes in hemoglobin concentrations associated with brain activity. Quantitative comparisons between fNIRS and fMRI have demonstrated the reliability of fNIRS either in tasks (36–38) or in resting-state (39–41). Recently, by using an optode arrangement with multiple channels designed to cover the main areas of the brain, the resting state fNIRS imaging has demonstrated its power in characterizing brain functional connectivity and brain network properties (42, 43). Compared with fMRI, fNIRS is easily operated in an ecologically relevant context due to its relative insensitivity to motion artifacts, making it especially suitable for naturalistic research in which participants are allowed to speak or move relatively freely. Thus, these combined findings demonstrate that the multiple-channel fNIRS imaging technique holds the potential to explore the task-evoked brain activation pattern at the whole-brain level.

In the present study, we adopted a multiple-channel fNIRS imaging technique to record cerebral hemodynamic activity from 56 right-handed adult participants while performing the picture-naming and the landmark tasks. The aim of this experiment was to examine the relationship of functional specification between language production and visuospatial attention. We hypothesize that: (1) typical brain activations of left-lateralized language production and right-lateralized visuospatial attention will be observed; (2) the negative correlation between language production laterality index and visuospatial attention laterality index would be observed on right-handers if the “causal” relationship was universally correct.

## Materials and Methods

### Participants

Fifty-six healthy students (34 females; 22 ± 2.02 years old; age range, 18–24 years old) from Beijing Normal University participated in the present study. The sample size met the requirements to achieve a power of 0.95 in a paired samples *t*-test at a threshold of *p* = 0.05 with medium effect (i.e., 0.5, *N* = 54), and a power of 0.8 in a two-way repeated-measures ANOVA at a threshold of *p* = 0.05 with medium effect (i.e., 0.2, *N* = 36). Justification of the sample size was checked with G^*^Power (44). In addition, we measured the head circumference of each participant (Mean ± SD: 56.89 ± 1.65). Considering the head circumference varied from 53.5 to 60 cm among adult participants, we estimated that measurement inconsistency across participants was in the range of 0.3 cm [i.e., (60–53.5)/60 × 3 cm; 3 cm is the S-D separation distance] (42, 45).

All participants had no history of neurologic, medical, and psychiatric problems. All participants were right-handed as assessed by the Edinburgh Handedness Inventory (75.29 ± 23.51) (46). Approval for this study was obtained from the Institutional Review Board of the State Key Laboratory of Cognitive Neuroscience and Learning, Beijing Normal University. The participants provided written informed consent before the initiation of the experiments.

### Tasks and Stimuli

To investigate the relationship of functional lateralization between language-dependent processing and visuospatial processing, we conducted a picture-naming task and a landmark task to identify the cerebral lateralization of language production and visuospatial attention, respectively. Each participant started with an ~20-min resting-state recording and then was instructed to complete the landmark and picture-naming tasks after a quick break. The task order was counterbalanced across participants to avoid the sequence effects. The experiment lasted for approximately 1 h and 15 min; participants were told to terminate the experiment whenever they felt uncomfortable. The experiment was carried out in a silent room with dim lighting. Participants were seated comfortably in a chair in front of a PC screen used for presentation (1,024 by 768-pixel resolution and 60 Hz refresh rate). The eye-to-screen distance was 107 cm. Participants were given ~2 min to adapt themselves prior to the experiment, during which the experimenter briefed them on the experimental procedure.

Finally, 54 participants completed the landmark task, and 52 participants completed the picture-naming task, of which 50 participants completed both.

#### Picture Naming Task

**Figure 1:**
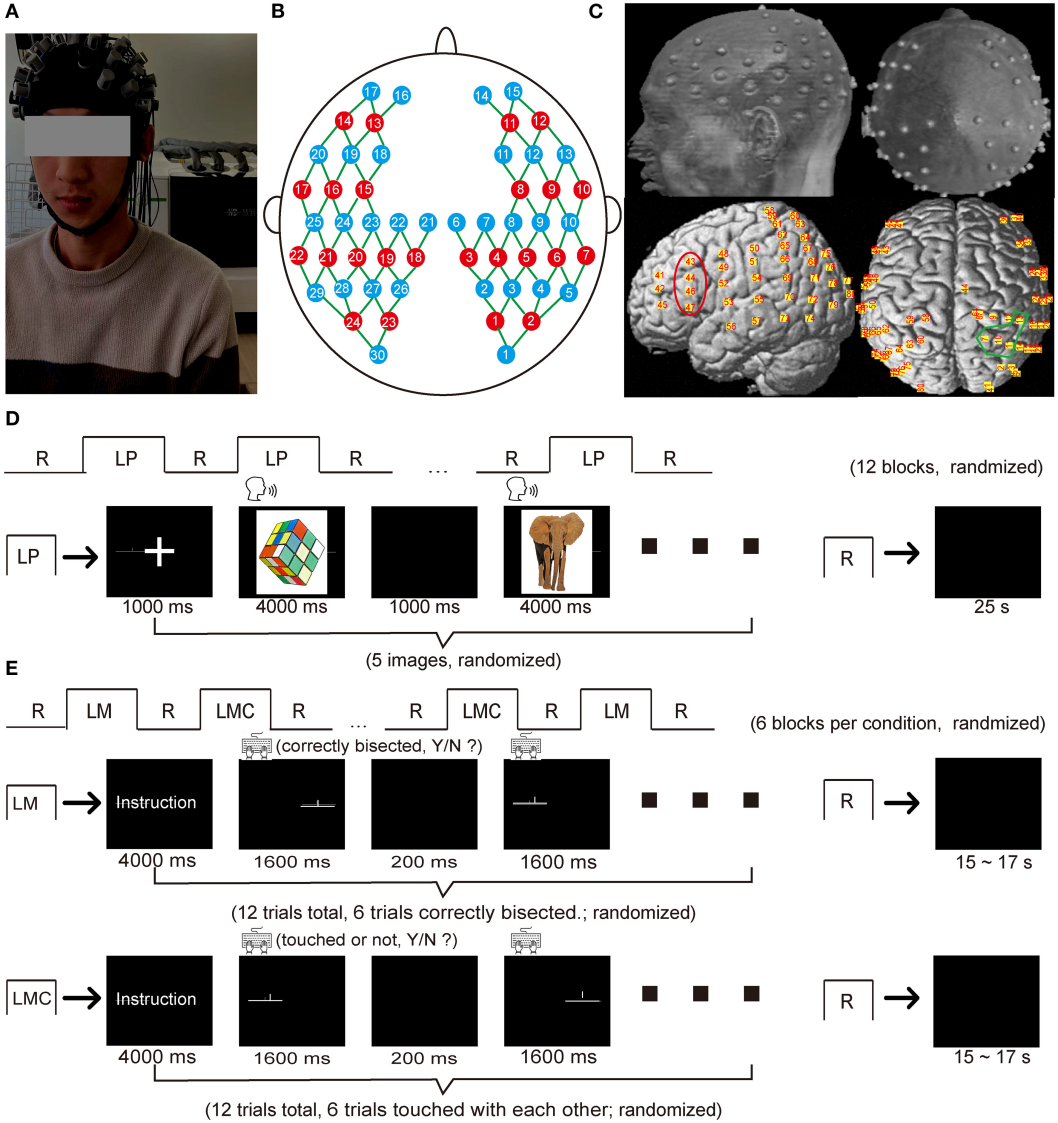
fNIRS optode arrangement and experimental protocol. **(A)** A photo obtained from a participant during the data acquisition. **(B)** A schematic of the fNIRS cap (22 sources: red circles, 30 detectors: blue circles) with 80 measurement channels (green line) covering the brain regions of frontal, temporal, parietal, and occipital lobes **(C)** The structural MRI image with the probe holder in which the gray dots represent the optode positions on the brain scalp, and the yellow dots represent the corresponding channel positions projected onto the brain surface using NIRS-SPM. The red area represented the typical regions activated during the language production task (i.e., the inferior frontal gyrus), and the blue area represented the typical regions activated during the spatial attention task (i.e., the inferior parietal sulcus and the superior parietal lobule). **(D)** Procedure of the picture-naming task. **(E)** Procedure of the landmark task. LP, language production (picture-naming task in this study); LM, landmark condition; LMC, landmark control condition; R, rest.

A block-designed picture-naming task was used to characterize language production (Figure 1D). The task consisted of 12 blocks. Each block consisted of 5 pictures followed by a 24 s rest in which a fixation instructed the participants to relax. Each picture was presented for 4 s in the center of the screen with 1 s interstimulus interval during the task. Participants were required to name the pictures as soon as they were presented. Of note, we chose picture naming rather than word generation for two reasons. Firstly, picture naming has similar speech production requirements as word generation (47). In addition, the picture superiority effect and production effect of picture naming task (48) are compatible well-with the advantage of fNIRS' high tolerance to artifacts, which allows the participants to speak relatively freely. Secondly, it is easy to use and to equate task difficulty across participants, which is consistent with one of the goals of the current study, namely to validate the performance of the multichannel fNIRS technique in simultaneously measuring spatially distant brain activations.

#### Landmark Task

A block-designed landmark task was used to characterize visuospatial attention (Figure 1E), which was essentially identical to the previous study (7). The task consisted of a landmark condition (LM) and a landmark control condition (LMC), and each condition comprised 6 blocks alternated with a 15–17 s rest block as baseline control. Each block started with an instruction lasting for 4 s and indicating which task was to be performed. Each active block consisted of 12 trials in a randomized order and lasted for 21.6 s. In the LM condition, each trial was composed of a horizontal line (15 cm long, a visual angle of 8°) and a short vertical line, in which the horizontal line was pre-bisected by the vertical line. The vertical line either precisely bisected the horizontal line (50% of the trials) or slightly deviated to the left or the right (the remaining 50% of the trials). Three deviation distances were used: 2.5, 5.0, or 7.5% of the horizontal line length. In the LMC condition, the stimuli structure was similar to the LM condition. Half of the trials in each block were modified so that the short vertical line was placed slightly above the horizontal line and did not contact it. Each trial was presented for 1.6 s with a 200 ms interstimulus interval. Participants were required to determine whether the vertical line bisected the horizontal line in the middle in the LM condition or whether the vertical line contacted the horizontal line in the LMC condition. Participants were instructed to press the “F” button on the keyboard when the vertical line bisected the horizontal line in the middle or did not contact the horizontal line; otherwise, they were instructed to press the “J” button instead.

### Data Acquisition

A continuous-wave near-infrared optical imaging system (Nirscan, Hui Chuang, China) (49) with a sample rate of 17 Hz was used to record the hemodynamic response for each participant (Figure 1). The optode array contained 24 light sources (740 and 850 nm) and 30 detectors, each with two wavelengths. The probes generated 80 measurement channels with a fixed 3 cm source-detector distance covering the brain's frontal, temporal, parietal, and occipital lobe regions (Figure 1B). According to the international 10–20 system, optodes were placed on participants' heads with the external auditory canals and vertex as the landmarks (Figure 1C).

### MRI Coregistration

We acquired structural MRI images from an arbitrarily chosen participant using a 3T Siemens Tim Trio MRI scanner at the Imaging Center for Brain Research, Beijing Normal University, to obtain the exact positions of the measurement channels on the brain surface. During MRI data acquisition, the participant lay supine while wearing the probe holder. The probe holder was pasted with Vitamin E capsules at positions representing the measurement channels (Figure 1C). T1-weighted structural images were acquired using a magnetization-prepared rapid gradient echo (MPRAGE) sequence: 176 slices, TR = 2,600 ms, TE = 3.02 ms, FOV = 256 × 224 mm 2, voxel size = 1 mm × 1 mm × 1 mm, flip angle = 8° and slice orientation = sagittal. The MR images were normalized into MNI space using the SPM8 software^1^, and the MNI coordinates for each channel were determined using NIRS-SPM (50) (See Supplementary Materials; Supplementary Table 1).

### Data Processing

We conducted two quality control steps for initial channel pruning: (1) Considering that oxygenated-hemoglobin signal (HbO) and deoxygenated-hemoglobin signal (HbR) are negatively correlated during cognitive tasks and motion artifacts may cause this negative correlation to become more positive, channels in which the correlation between HbO and HbR is equal to −1 or above 0.5 were considered bad channels (51); (2) channels without cardiac component (~1Hz) in the power spectrum were also marked as bad channels. Participants with more than 20% noise channels were excluded from the further analysis (52). As a result, 5 and 3 participants were separately removed from the landmark task and the picture-naming task, and the average channel remained for both tasks was 81.51% (*N* = 65) and 88.75% (*N* = 72), respectively.

For the remaining data, we conducted the following steps to obtain the HbO and HbR using Homer 2 software (53): (1) converted the raw intensity signals into optical density (OD); (2) corrected motion artifacts using the spline-SG method on OD, a combination of Spline interpolation method and Savitzky–Golay filtering (frame = 6 s, *p* = 0.99) (Supplementary Figure 1) (54); (3) bandpass filtered (0.008–0.5 Hz) the OD to remove the low-frequency drift and high-frequency noise; (4) calculated the hemoglobin changes of the HbO and HbR according to the modified Beer-Lambert law. To this end, we performed principal component analysis (PCA) to identify and remove systemic physiological noises. These systemic fluctuations are thought to have a stronger spatial covariance structure than the task-based signal and are associated with the arterial pulse, respiration, and heartbeat, thus contaminating the actual cortical response. Specifically, a separate resting-state data file (5 min in this case) was first used to derive the principal components, then the first few components (2 components in this case) were projected from the task-based data to remove the strongly spatially correlated physiological noise. This procedure was performed separately for HbO and HbR data (55).

Next, we rejected the suspicious blocks according to the following criteria: (1) the blocks in which signal variations were larger than 0.1 mM x mm throughout 0.2 s (56); (2) the blocks with accuracy < 0.5 according to behavior performance. After removing the suspicious blocks, participants with <3 blocks were excluded from further analysis (57). Accordingly, no participants were removed at this stage, and the average block for the picture-naming task was 8.85 ± 1.80, and for the landmark task was 3.95 ± 1.7. Consequently, a linear detrend for each of the non-rejected blocks and then a baseline correction on the 5 s preceding onset of the block were performed. Finally, we averaged the blocks' hemodynamic responses (−4 to + 40 s) by the condition in each channel for each participant.

Here, we focused on HbO since its higher signal-to-noise ratios than the HbR signal in fNIRS data (41). To obtain the mean hemodynamic response for statistical purposes, we selected 30-s data points in the middle of each block-averaged hemodynamic response-and then averaged them.

### Statistical Analysis

#### Behavioral Performance

To evaluate the performance of landmark task in measuring visuospatial attention, we first compared the differences in reaction time and reaction accuracy between the LM condition and the LMC condition using paired *t*-test. Next, a two-way repeated-measures ANOVA was applied to the reaction time and reaction accuracy of the LM condition to evaluate the unilateral neglect effect of the landmark task in terms of error rate, with the hemispheres (left vs. right) and deviation distances (2.5, 5.0, and 7.5%) as two within-subject factors. In addition, we also examined whether the laterality indices were associated with behavioral performance (1).

#### Brain Activation Identification

After calculating the mean hemodynamic response for each channel, we conducted a simple *t*-test on the mean responses across participants per channel to examine the local brain activation strength. The resulting *t*-values of each channel were then projected back to the brain surface to evaluate the brain activation patterns of each task across the whole brain, in which an extremum voxel method was used for rendering (58).

To enhance statistical power and calculate the laterality index of each task in subsequent analysis, we further chose a target region of interest (ROI) for each task. Regions of interest were selected based on previous studies. In specific, we selected our region of interest to a relatively larger area covering the typical regions responsible for the language production (i.e., IFG in the current study) and spatial attention (i.e., IPS/SPL in the current study) according to previous studies (7, 19). Channels located in these areas were determined by the possibility given by NIRS_SPM according to the Brodaman Anatomical Labeling template (50). Eventually, we selected channels 43, 44, 46, and 47 in the left hemisphere and their homologs (channel 34, 35, 38, and 39) in the right hemisphere as the ROI for the picture-naming task. Similarly, we selected channels 60, 62, 63, and 64 and their homologs (channel 7, 10, 11, and 13) as the ROI for the landmark task (Figure 1C). To this end, the mean hemodynamic responses were calculated across the ROI. Next, the mean hemodynamic response over each ROI was calculated. The resulting data were then subjected to a statistical model (i.e., pair *t*-test or two-way repeated-measures ANOVA) to evaluate the hemispheric activation difference.

#### Hemispheric Lateralization

To investigate the degree of hemispheric lateralization on the tasks of picture-naming and landmark, we calculated the laterality index (*LI*) using the following equation: *LI* = *(L* – *R)/(L* + *R)*, where *L* and *R* represent the mean hemodynamic change within the ROI in the left and right hemispheres, respectively. The *LI* ranges from −1 to 1, with a positive value of LI representing a leftward specification and vice versa. Furthermore, to evaluate the relationship of functional lateralization between language production and visuospatial attention, we calculated the Pearson correlation on the laterality index between picture-naming task and landmark task across all participants.

## Results

### Behavioral Performance of the Landmark Task

**Figure 2:**
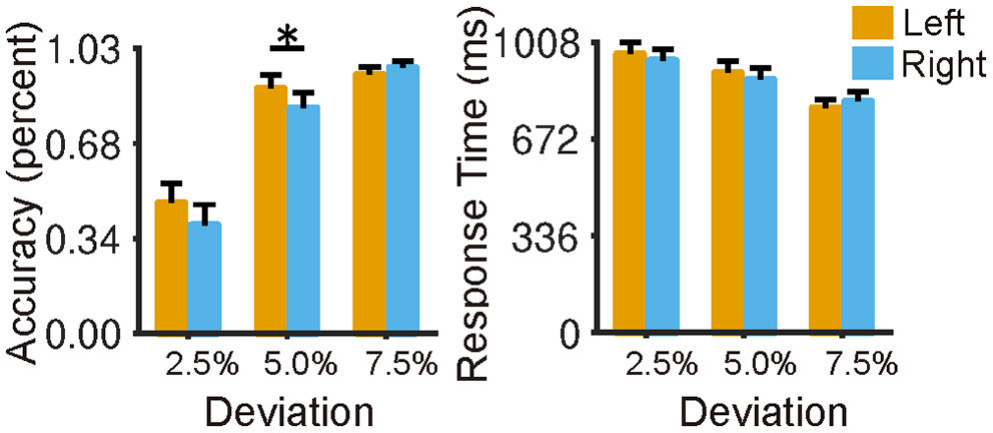
Behavioral performance of the landmark task in terms of reaction time and accuracy. Participants showed a slight leftward bias in the landmark condition (2.5, 5, 7.5% indicate the bias degree to the midpoint of the horizontal line), and no speed-accuracy trade-off was observed. The error bar represents a 95% confidence interval. *p < 0.05, **p < 0.01, ***p < 0.001.

The participants showed a slight leftward bias in the LM condition (Figure 2), with higher accuracy as the deviation pointed to the left of the center (accuracy for 2.5% condition: 46.9 ± 26.3%; accuracy for 5% condition: 88.2 ± 18.4%) than that to the right of the center (accuracy for 2.5% condition: 38.9 ± 28.1%; accuracy for 5% condition: 81.2 ± 20.4%). A two-way repeated-measures ANOVA (left and right vs. 2.5, 5, and 7% deviation) revealed a main effect of deviation [*F*_(2,144)_ = 231.48, *p* < 0.0001] and an interaction effect [*F*_(2,144)_ = 4.25, *p* = 0.016)]. *Post-Hoc* indicated that the left bias was prominent at the location of 5% to the center (*t* = 2.28, *p* = 0.026). Furthermore, as expected, we found that participants' performance in the LMC condition was better than that in the LM condition (Supplementary Figure 2, left). Specifically, they made ~7% errors on average in the LMC condition compared with 20% in the LM condition (*t* = 12.73, *p* < 0.001), and they were also faster in the response time in the LMC condition (response time = 802 ms) than that in the LM condition (response time = 883 ms) (Supplementary Figure 2, right) (*t* = −5.94, *p* < 0.001). The results suggested that participants needed to make more efforts to meet the requirements of the attention-focused task. In addition, we found a significant correlation (*r* = −0.34, *p* < 0.01) between overall performance and laterality index in terms of response accuracy at a deviation of 2.5%, suggesting that the brain lateralization is associated with behavioral performance.

### Identification of Brain Activation Pattern

**Figure 3:**
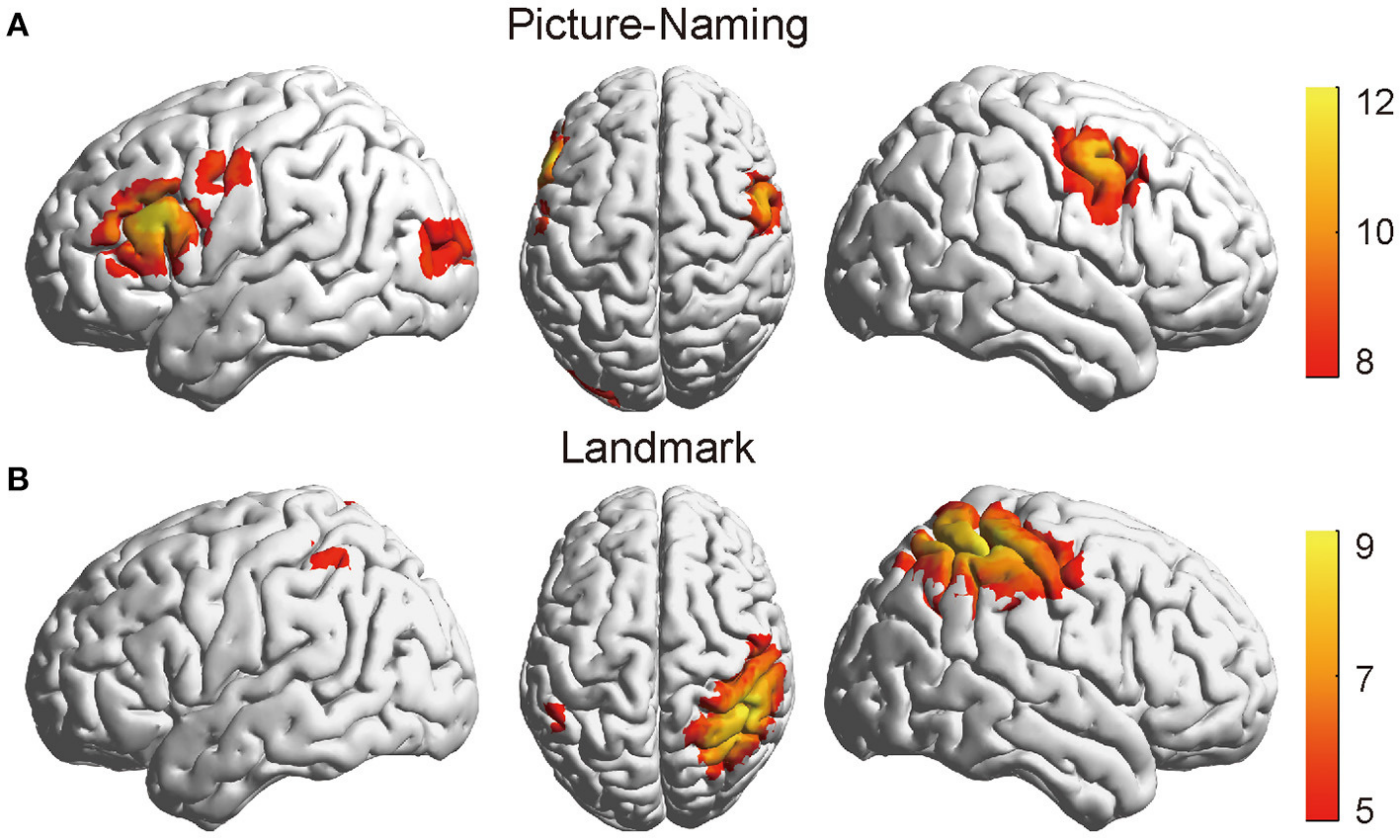
The thresholded activation t-maps of the picture-naming and landmark tasks. **(A)** The brain showed strong activation in the IFG area of the left hemisphere in the picture-naming task (FDRBH corrected, *q* < 0.05). **(B)** The brain showed increased activation in the IPS/SPL of the right hemisphere in the landmark task (FDRBH corrected, *q* < 0.05). IFG, left inferior frontal gyrus; IPS/SPL, inferior parietal sulcus, and the superior parietal lobule areas.

Figure 3 shows the thresholded activation t-maps for picture-naming (Figure 3A) and landmark tasks (Figure 3B), respectively. Multiple comparisons were corrected using false discovery rate correction approach (FDR) (59), Specifically, the Benjamini-Hochberg correction method was used (60). For the picture-naming task, strong brain activation was found in the left inferior frontal gyrus (IFG) and the bilateral supplementary motor areas (SMA) (FDR_BH_ corrected *p* < *0.05*). For the landmark task, strong brain activation was found in the right inferior parietal sulcus and the superior parietal lobule areas (IPS/SPL) (FDR_BH_ corrected *p* < *0.05*). Compared with the control condition (LMC) (Supplementary Figure 3), the LM task brought about a stronger and more asymmetrical activation pattern between hemispheres. Similar activation patterns of both tasks can be seen after randomly dividing the data into two subsets and then conducting the same analysis on each subset (Supplementary Figure 4). These identified activation regions were consistent with those found in previous studies (7, 19). They demonstrated the validity of fNIRS-derived activation detection in measuring language production and visuospatial attention.

### Comparison of Hemodynamic Response Between Hemispheres

The mean hemodynamic response was calculated across the regions responsible for language production (i.e., IFG) and visuospatial attention (i.e., IPS/SPL). Specifically, channels 43, 44, 46, and 47 within the left hemisphere and their homologs within the right hemisphere (i.e., channels 34, 35, 38, and 39) were identified as the regions of interest for the picture-naming task. Similarly, channels 60, 62, 63, and 64 within the right hemisphere and their homologs within the left hemisphere (i.e., channels 7, 10, 11, and 13) were identified as the regions of interest for the landmark task. These regions were also the most activated regions in the current study for each task.

**Figure 4:**
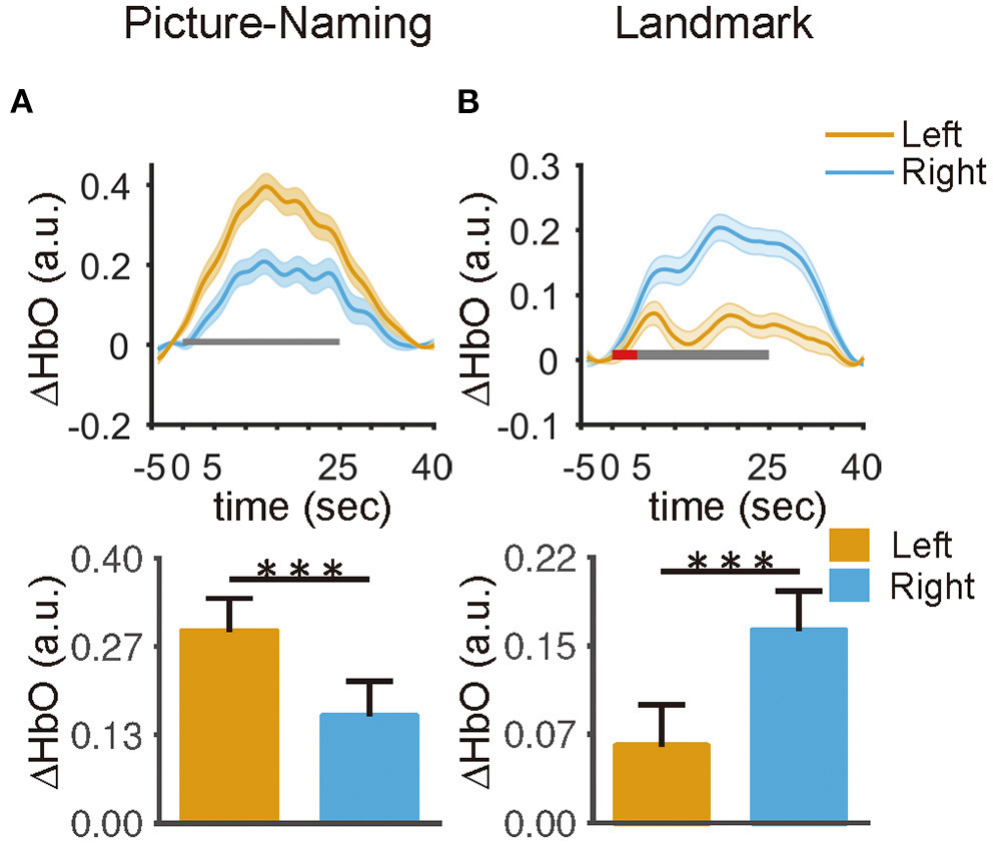
The hemispheric differences in the averaged hemodynamic responses within the ROIs. **(A)** The left ROI exhibited a larger response than the right ROI in the picture naming task (*t* = 5.3, *p* < 0.001). **(B)** The right ROI exhibited a larger response than the left ROI under the landmark condition in the landmark task (*t* = 6.6, *p* < 0.001). The red and black rectangle represents the duration of the instruction and task period, respectively. The error bar represents a 95% confidence interval. *p < 0.05, **p < 0.01, ***p < 0.001.

For the picture-naming task, the mean oxy-hemoglobin concentration showed an obviously larger increase in the left hemisphere than that in the right hemisphere (paired *t*-test, *t* = *5.31, p* < *0.01*) (Figure 4A). For the landmark task, a two-way win effect of hemispheres [*F*_(1,46)_ = *26.44, p* < *0.0001*] and an interaction effect between hemisphere and task condition [*F*_(1,46)_ = *9.54, p* < *0.01*]. Tukey HSD revealed that participants exhibited a significantly larger mean hemodynamic change in the right hemisphere than that in the left hemisphere in the LM condition (*t* = –*6.69, p* < *0.001*) (Figure 4B), and no significant difference was found in the LMC condition (*t* = –*1.92, p* = *0.14*) (Supplementary Figure 5). In addition, results derived from HbR were also reported (61) (Supplementary Figure 6). These findings demonstrated a hemisphere bias of the brain in processing language production and visuospatial attention.

### Quantitative Calculation of Functional Lateralization

**Figure 5:**
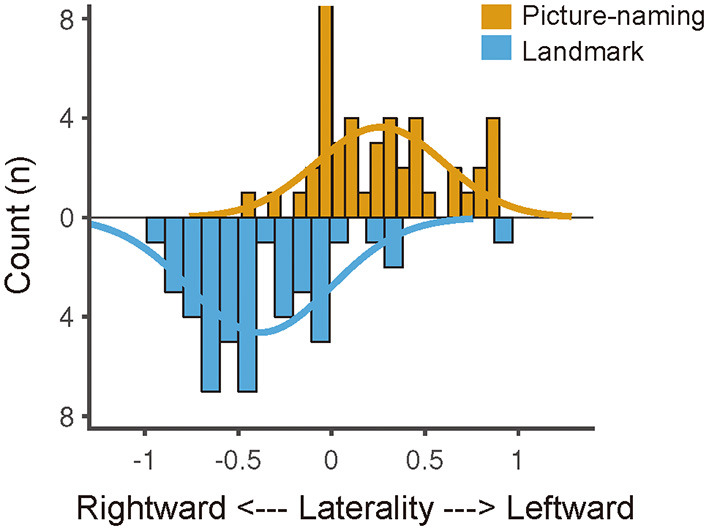
Laterality index distribution of the picture-naming and landmark task. The laterality index distribution was fitted by Gaussian curves, suggesting a left-lateralized distribution of the picture-naming task and a right-lateralized distribution of the landmark task. The difference between the two distributions was significant (*p* < 0.001, Kolmogorov–Smirnov test).

Figure 5 shows the distribution of functional lateralization from all 45 participants who completed both tasks. For the picture-naming task, most of the participants (*N* = *32*) showed typical left-lateralized activation pattern (*LI* > *0*), and the laterality index was larger than 0 (*t* = *5.14, p* < *0.001*). For the landmark task, most of the participants (*N* = *40*) showed typical right-lateralized activation pattern (*LI* < *0*), and the laterality index was smaller than 0 *(t* = –*6.78, p* < *0.001*). These two tasks showed an obvious difference in the distribution of functional specification (Kolmogorov-Smirnov tests, *p* < *0.001*).

### The Relationship of Functional Lateralization Between Language Production and Visuospatial Attention

**Figure 6:**
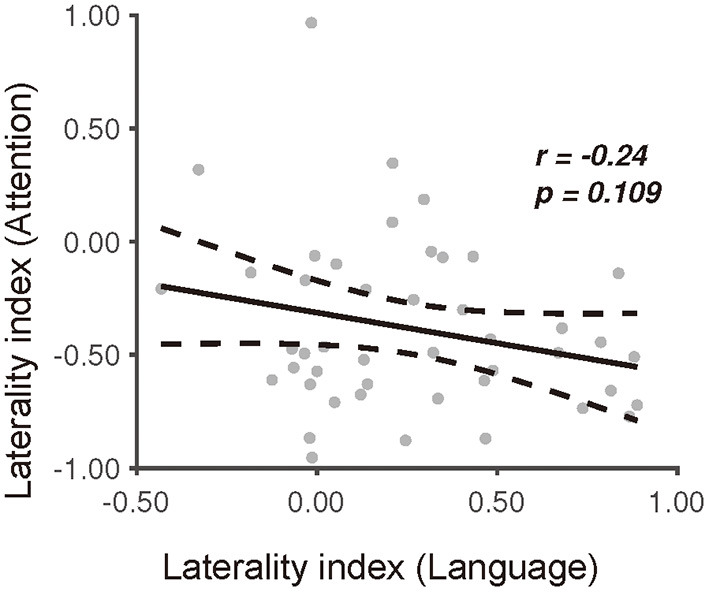
The relationship between the picture-naming and landmark task in terms of the degree of laterality index. Dissociation of the laterality index between the picture-naming task and the landmark task.

To reveal the mutual relationship of functional lateralization between language production and visuospatial attention, we applied a Pearson correlation analysis to the *LI* between these two tasks. It was found that the degree of the picture-naming laterality index had no significant correlation with that of the landmark laterality index (*r* = −*0.24, p* = *0.109*) (Figure 6). Furthermore, the linear trend disappeared after we excluded the outliers in the data identified with the regstats function (*r* = *0.01, p* = *0.94, N* = *39*) (MATLAB, 2019a). These results indicated an independent process of functional lateralization of language production to that of visuospatial attention in right-handers. In addition, results derived from HbR were also reported (61) (Supplementary Figure 7).

## Discussion

Previous findings regarding the relationship between language-dependent processing and visuospatial processing are inconsistent, with some supporting the independent hypothesis and some supporting the causal hypothesis. Here, we examined these two hypotheses with a group of right-handers using multichannel fNIRS imaging. We adapted a picture-naming task and landmark task to measure the cerebral lateralization of language production and visuospatial attention. We found a typical brain activation pattern favoring the left hemisphere for language production and the right hemisphere for visuospatial attention. Although most participants displayed a typical complementary specialization, the relationship of functional specification revealed by language production and visuospatial attention did not show a significant correlation. These results and their implications are discussed in turn.

As expected, the picture-naming task predominantly activated the left hemisphere, while the landmark task predominantly activated the right hemisphere in our study. These findings were consistent with previous findings in right-handers (18, 20), illustrating that functional lateralization is a fundamental principle of brain organization. From a computational benefit point of view, functional lateralization can be considered a particular case of functional specialization of the brain (25). The main idea is to divide the larger functions into small independent processes to ensure the information processing is more effective and efficient. Here we refer to it as the “computational benefit hypothesis.” Such a point has been proven across a variety of species (1, 62). Recently, researchers have proposed three possible mechanisms linking such an asymmetrical organized neural system to performance: (1) increase in neural processing capacity by carrying out simultaneous processing (62); (2) avoidance of redundancy of neural networks (63); (3) reduction of intra-hemispheric time conduction delay (64). Furthermore, the prevalence of left hemisphere dominance for language production and right hemisphere dominance for visuospatial attention in the population is compatible well-with the “intraspecies evolutionary stable strategy” hypothesis proposed for animal models (65). It was suggested that the formation of population-level asymmetries involves two steps (66). In step one, individual-level asymmetries were formed because of the advantages of such an asymmetrical neural organization in practice. Then, population-level asymmetries were gradually formed during evolution due to the benefit of cooperation against the predator at the synergistic level (i.e., typical). However, a reversed asymmetry would also arise in the minority at the antagonistic level because such population bias could also be learned by their predators and enhance the predators' ability to anticipate their targets' behaviors (i.e., atypical). Thus, the functional lateralization of language production and visuospatial attention might benefit from evolutionary advantages for selection pressure and adaptive processes.

While the population bias regarding language production and spatial attention prevails in most individuals, a central question underlying its ontogenesis remains as to whether language production would force spatial attention to be located in a separate hemisphere. Accordingly, the crowding hypothesis and independent hypothesis were proposed. The former suggests a competition mechanism for limited neural resources within the same hemisphere, indicating that language production and spatial attention would lateralize oppositely to avoid such a competition (25, 26). By contrast, the latter suggests a mechanism of possibility, contributing functional lateralization to a consequence of many independent sources (17, 27). Here, we revealed a dissociation between picture-naming task and landmark task, providing compelling evidence for the independent hypothesis dominant at the moment (23). Based on the computational benefit hypothesis, the independent hypothesis may represent a mechanism in which two cognitive processes spatially distributed may share fewer neural resources and avoid the competition of limited neuronal capacity, thus maximizing the information processing efficiency. In this case, two functions are processed in isolation to a large extent, thus yielding little correlation. A recent study revealed two distinct circuits of visual word form area (VWFA) involved in integrating language and attention (67). That is, the structural connectivity linking VWFA to lateral temporal language network correlated with language but not with visuospatial attention, while connectivity linking VWFA to dorsal frontoparietal attention network correlated with visuospatial attention but not with language. The authors concluded that these findings might represent an early dissociation of language production with spatial attention at the perceptual level. Therefore, it may indicate that right-handers possess a mechanism in which language production and spatial attention work separately.

However, previous findings also revealed a significant correlation between language production and spatial attention, especially in left-handers, supporting the causal hypothesis (7, 18). The robust correlation between language lateralization and handedness has been well-documented (29, 30). Accordingly, if language production causally interacts with spatial attention, we would expect a stronger correlation between language production and spatial attention in right-handers because the population level bias for both cognitive functions is more pronounced in right-handers than in left-handers. However, our finding was against their results, implying that the interactive neural pattern of language production and spatial attention differed in left-handers. Firstly, our results were in line with those found with right-handers (20, 28). Secondly, it also helped to reconcile the discrepancies of weak (19) or no correlation (17) between language production and spatial attention in studies including both right and left-handers. In a recent study of 142 right-handers and 151 left-handers, researchers discovered a significant correlation between language production and spatial attention in left-handers only but not in right-handers or mixed-handers, confirming the discrepancy of the pattern between left- and right-handers (18). Thus, the question is what mechanism underlies such a discrepancy. Here, we speculated another mechanism in left-handers, which supported the causal hypothesis and did not violate the principle of the computational benefit hypothesis. To maximize the use of the limited neural resources of the brain, two cognitive processes having common cognitive components could share a common neural network to avoid neural redundancy. In this case, the common cognitive component would link the two functions together, yielding a modest correlation depending on the proportion of the shared component. For example, the well-documented relationship between language and handedness (29) could be an example supporting such a mechanism. Although they are distinct functions, the motor coordination for speech (i.e., mouth and tongue) is similar to that for writing and drawing (i.e., fined movements) (28). Recently, more evidence has been manifested (67, 68). For instance, in a recent study investigating the mechanisms underlying the control of working memory and attention, the authors revealed that the prefrontal cortex showed a similar neural representation for the selection process of working memory and attention, indicating a role of the prefrontal cortex as a domain-general controller (68). Thus, there may exist a common neural network that links language production and spatial attention in left-handers.

Further, we speculated two possible mechanisms underlying the interaction between language production and spatial attention, which depend on handedness. However, these two mechanisms converge so that the shared neural network determines the neural correlations of two lateralized functions. So, what exactly is the shared neural resource? Some researchers proposed the maintenance ability of the inhibition mechanism of the corpus callosum (CC) (18). The inhibition mechanism of CC refers to CC's ability to inhibit homologous brain activation, thereby facilitating the hemisphere specialization, such as language production and spatial attention. The authors suggested that this mechanism remains active in left-handers but may reach a stable pattern in right-handers and not play anymore. Such an interpretation can also explain the large variability of complementary specialization patterns in left-handers but not in right-handers. However, we proposed that CC's inhibition mechanism could provide less interpretation for the co-lateralization of language production and spatial attention to the same hemisphere (16). Thus, we suggested that further work should focus on the similarities or discrepancies of the brain activation patterns evoked by language production and spatial attention in left-handers to detect the areas serving both cognitive processes. Novel paradigms like dual-task (68) and new data analysis direction (67) may help us find these potential neural circuits that differentiate left-handers from right-handers. As suggested in Ref. 8, the intrahemispheric pattern of activation, not the interhemispheric side, is the crucial feature for neural processes. To sum up, these findings indicate that handedness is an essential factor that we should consider when examining the mechanism of complementary specialization.

The current study observed a task-evoked brain activation pattern comparable to previous fMRI studies (8, 19). To the best of our knowledge, this is the first study using the multichannel designed fNIRS technique to simultaneously detect two kinds of brain activation that are spatially distant It facilitates a broad application of this technique to examine brain activation patterns at the whole-brain level. Moreover, our results also highlighted the ecological validity of the fNIRS technique. As shown in Figure 1, participants showed a leftward bias in terms of error rate in the landmark condition, reflecting a right hemisphere attention bias to the left-field and indicating consistency between behavioral performance and brain activation. However, such consistency was not observed in previous studies with fMRI (7, 69). These studies observed a rightward bias in behavioral performance regardless of handedness when participants were in the scanner conducting the same task. They attributed such an effect to the far space experience of the stimulus. That is, stimuli positioned outside the participants' reaching space would alter participants' behavioral performance (69). As a result, the landmark task may not be completely the same as in front of the computer when the stimulus was 1.1 m away from the eye in the scanner. In addition, they speculated that such an influence might be the reason behind the extra bilateral activation in the occipital and occipitotemporal regions (70). Thus, we demonstrated that fNIRS complemented fMRI in measuring spatial attention in an ecologically relevant context without being affected by far space experience.

Finally, investigating the relationship between lateralized functions might also provide us with a new way to relate functional lateralization to cognitive performance. Consistent with previous results (1), we found that the laterality index of the landmark task correlated significantly with the individual's overall performance. While functional lateralization is thought to be a biomarker of human development (10), most studies focus on a single function rather than their mutual relationship (3, 4). However, recent studies investigating the relationship between two lateralized functions have demonstrated that the lateralization pattern of two or more functions, rather than the lateralization of a specific function, seems more informative in evaluating human development as a biomarker. For example, researchers suggested that participants whose language and spatial attention lateralized to the same hemisphere decreased in their verbal comprehension and perceptual organization ability compared to those whose language dissociated with spatial attention in a separated hemisphere (21). Recently, in a study relating functional asymmetry patterns of individuals to behavioral performance, the authors revealed that the atypical lateralization of a certain function did not lower the participants' average performance on measures of intelligence and general cognition. Instead, participants with two or more atypical lateralized functions performed worse on a neuropsychological test battery than those with typical or mirror-reversed function lateralization patterns (9). These findings suggested that the patterns of functional asymmetries seem to be more informative about an individual's cognitive ability. However, it is unclear how these functional lateralization patterns emerge and develop with age. In this regard, our work demonstrates the potential use of the multichannel designed fNIRS technique for brain developmental research, extending the populations to larger cohorts of subjects.

There were several limitations in the current study. (1) The word generation task outperforms picture naming in detecting brain activation of language production (71). Thus, a quantitative comparison between word generation and picture-naming in further work is needed to improve the reliability of our results. (2) Variability of head anatomy between subjects may bias our results and make the calculation of the laterality index biased; further studies equipped with a 3D digitizer is needed for validation. (3) Monte Carlo (MC) simulation in fNIRS studies promises to improve accuracy by examining light propagation in brain areas (72). Therefore, further studies will consider incorporating this approach to make the results more accurate. (4) The removal of systemic physiological noises is challenging. Although the PCA approach performed well in the current study (55), recent studies found that the short-separation channels approach outperformed it when multiple short-separation channels were used (73). We speculated that a better global noise removal approach could improve the performance of fNIRS in detecting brain activations. Thus, further studies equipped with multiple short-separation channels approach is needed to validate our findings. (5) Finally, the frequency information plays an essential role in assessing the relationship between cerebral asymmetries (74). If the spatial attention lateralization is causally related to language lateralization, one would expect a reduced frequency of the typical bias in non-right handers, comparable to the frequency difference between right and left-handers in the language-related task (75). Further work including sufficient left-handed participants will provide further evidence for the relationship between language production and spatial attention.

## Conclusion

This study examines the correlation relationship between language production and visuospatial attention on a group of right-handers. Presumably, right-handers will show a stronger association between these two lateralized functions, given their overall lower phenotypic variability and stronger population-level lateralization biases than left-handers.

However, our results were against our assumption and demonstrated a dissociation between these two functions. We concluded that the complementary pattern between language production and visuospatial attention is not completely obligatory but subject to handedness and speculated the existence of a distinct neural circuit linking language production to visuospatial attention in left-handers.

## Data Availability Statement

The raw data supporting the conclusions of this article will be made available by the authors, without undue reservation.

## Ethics Statement

The studies involving human participants were reviewed and approved by Institutional Review Board of the State Key Laboratory of Cognitive Neuroscience and Learning, Beijing Normal University. The patients/participants provided their written informed consent to participate in this study.

## Author Contributions

GJ and HN designed this study. GJ and GL collected the data. GJ analyzed the data and drafted the manuscript. HN revised the manuscript. All authors contributed to the article and approved the submitted version.

## Funding

This study was supported by the National Natural Science Foundation of China (81761148026 and 81571755).

## Conflict of Interest

The authors declare that the research was conducted in the absence of any commercial or financial relationships that could be construed as a potential conflict of interest.

## Publisher's Note

All claims expressed in this article are solely those of the authors and do not necessarily represent those of their affiliated organizations, or those of the publisher, the editors and the reviewers. Any product that may be evaluated in this article, or claim that may be made by its manufacturer, is not guaranteed or endorsed by the publisher.

## Footnotes

^1^ http://www.fil.ion.ucl.ac.uk/spm
