# Supplementary material for: Hemispheric Lateralization of Visuospatial Attention Is Independent of Language Production on Right-Handers: Evidence From Functional Near-Infrared Spectroscopy
Source: Front Neurol. 2022 Jan 14;12:784821. doi: 10.3389/fneur.2021.784821 (PMC8795708; doi:10.3389/fneur.2021.784821)

**Supplementary Materials**

**Key results from HbR**

**Hemispheric differences in changes of deoxy-hemoglobin concentration**

Mean changes in deoxy-hemoglobin concentration (HbR) were also calculated within each ROI. The left hemisphere showed a larger decrease than the right hemisphere (paired t-test, t = -2.59, p < 0.05) in the picture-naming task (Figure S5A). For the landmark task, a two-way repeated-measures ANOVA revealed a main effect of hemispheres (F (1,48) = 8.08, p < 0.01) and an interaction effect between hemisphere and task condition (F (1,48) = 5.84, p < 0.01). Tukey HSD revealed that the right hemisphere showed a greater decrease than the left hemisphere in the LM condition (t = 3.79, p < 0.001), while no hemisphere difference was found in the LMC condition (t = 0.76, p = 0.45) (Figure S5B). Similar to the findings with HbO, these findings manifested the hemispheric differences in response to language production and visuospatial attention, respectively.

**The relationship of functional lateralization between language production and visuospatial attention**

We applied a Pearson correlation analysis to the laterality indices derived from HbR between these two tasks and found no significant correlation (r = −0.03, p = 0.86) (Figure S7), similar to the findings derived from HbO.

**Tables**

**Table S1 The MNI coordinates and anatomical labels** **corresponding to the measurement channels**

| Channel | MNI coordinates | AAL Template | Lobes | Channel | MNI coordinates | AAL Template | Lobes |
| --- | --- | --- | --- | --- | --- | --- | --- |
| 1 | 24 -98 24 | Occipital_Sup_R | Occipital | 41 | -41 48 28 | Frontal_Mid_L | Frontal |
| 2 | 33 -82 45 | Occipital_Sup_R | Occipital | 42 | -47 48 18 | Frontal_Mid_L | Frontal |
| 3 | 41 -83 35 | Occipital_Mid_R | Occipital | 43 | -52 24 38 | Frontal_Mid_L | Frontal |
| 4 | 27 -102 12 | Occipital_Sup_R | Occipital | 44 | -56 24 26 | Frontal_Inf_Tri_L | Frontal |
| 5 | 44 -86 22 | Occipital_Mid_R | Occipital | 45 | -50 47 5 | Frontal_Inf_Tri_L | Frontal |
| 6 | 46 -88 8 | Occipital_Mid_R | Occipital | 46 | -59 24 16 | Frontal_Inf_Tri_L | Frontal |
| 7 | 22 -56 74 | Parietal_Sup_R | Parietal | 47 | -58 25 4 | Frontal_Inf_Tri_L | Frontal |
| 8 | 17 -35 80 | Postcentral_R | Parietal | 48 | -59 -3 44 | Precentral_L | Frontal |
| 9 | 29 -37 75 | Postcentral_R | Parietal | 49 | -64 -2 33 | Precentral_L | Frontal |
| 10 | 32 -59 70 | Parietal_Sup_R | Parietal | 50 | -62 -27 48 | SupraMarginal_L | Parietal |
| 11 | 39 -61 62 | Parietal_Sup_R | Parietal | 51 | -67 -27 38 | SupraMarginal_L | Parietal |
| 12 | 39 -39 70 | Postcentral_R | Parietal | 52 | -66 -3 22 | Postcentral_L | Parietal |
| 13 | 49 -41 61 | Parietal_Sup_R | Parietal | 53 | -66 -6 9 | Temporal_Sup_L | Temporal |
| 14 | 49 -64 53 | Angular_R | Temporal | 54 | -69 -28 26 | SupraMarginal_L | Temporal |
| 15 | 55 -65 43 | Angular_R | Temporal | 55 | -70 -30 9 | Temporal_Mid_L | Temporal |
| 16 | 59 -43 54 | Parietal_Inf_R | Parietal | 56 | -68 -7 -11 | Temporal_Mid_L | Temporal |
| 17 | 64 -44 46 | SupraMarginal_R | Parietal | 57 | -71 -29 -5 | Temporal_Mid_L | Temporal |
| 18 | 59 -66 29 | Angular_R | Temporal | 58 | -22 -38 76 | Postcentral_L | Parietal |
| 19 | 60 -67 13 | Temporal_Mid_R | Frontal | 59 | -34 -40 72 | Postcentral_L | Parietal |
| 20 | 68 -44 31 | SupraMarginal_R | Temporal | 60 | -26 -58 72 | Parietal_Sup_L | Parietal |
| 21 | 70 -46 14 | Temporal_Sup_R | Temporal | 61 | -41 -43 67 | Postcentral_L | Parietal |
| 22 | 62 -65 0 | Temporal_Mid_R | Temporal | 62 | -52 -47 57 | Parietal_Inf_L | Parietal |
| 23 | 71 -45 0 | Temporal_Mid_R | Temporal | 63 | -33 -61 66 | Parietal_Sup_L | Parietal |
| 24 | 61 -21 52 | Postcentral_R | Parietal | 64 | -43 -66 56 | Parietal_Inf_L | Parietal |
| 25 | 67 -22 42 | SupraMarginal_R | Parietal | 65 | -58 -49 50 | Parietal_Inf_L | Parietal |
| 26 | 57 4 47 | Precentral_R | Frontal | 66 | -63 -50 40 | SupraMarginal_L | Parietal |
| 27 | 63 5 37 | Precentral_R | Frontal | 67 | -50 -69 48 | Angular_L | Parietal |
| 28 | 70 -20 31 | SupraMarginal_R | Parietal | 68 | -53 -71 38 | Angular_L | Parietal |
| 29 | 72 -22 12 | Temporal_Sup_R | Temporal | 69 | -66 -51 26 | SupraMarginal_L | Temporal |
| 30 | 67 5 24 | Precentral_R | Frontal | 70 | -67 -53 12 | Temporal_Mid_L | Temporal |
| 31 | 67 3 13 | Rolandic_Oper_R | Frontal | 71 | -55 -74 26 | Angular_L | Temporal |
| 32 | 73 -22 -3 | Temporal_Mid_R | Temporal | 72 | -58 -72 10 | Temporal_Mid_L | Temporal |
| 33 | 67 0 -8 | Temporal_Sup_R | Temporal | 73 | -68 -50 -3 | Temporal_Mid_L | Temporal |
| 34 | 49 31 41 | Frontal_Mid_R | Frontal | 74 | -59 -70 -4 | Occipital_Inf_L | Occipital |
| 35 | 54 32 30 | Frontal_Inf_Tri_R | Frontal | 75 | -36 -83 44 | Occipital_Mid_L | Occipital |
| 36 | 37 52 32 | Frontal_Mid_R | Frontal | 76 | -40 -86 34 | Occipital_Mid_L | Occipital |
| 37 | 44 54 21 | Frontal_Mid_R | Frontal | 77 | -21 -99 25 | Occipital_Sup_L | Occipital |
| 38 | 59 32 17 | Frontal_Inf_Tri_R | Frontal | 78 | -44 -88 21 | Occipital_Mid_L | Occipital |
| 39 | 59 32 4 | Frontal_Inf_Tri_R | Frontal | 79 | -47 -88 6 | Occipital_Mid_L | Occipital |
| 40 | 47 55 9 | Frontal_Mid_R | Frontal | 80 | -26 -102 14 | Occipital_Mid_L | Occipital |

The most likely anatomical labels are shown. Anatomical labels were determined using the Automated Anatomical Labeling (AAL) (Tzourio-Mazoyer et al., 2002). All values are in millimeters. Abbreviations: L = Left hemisphere, R = Right hemisphere, Sup = Superior, Mid = Middle, Inf = Inferior.

**Figure** **Captions:**

**Figure S1**. The fNIRS signals before and after motion correction using the SpinleSG approach. (Left) An example of the optical density time courses at 740 nm. For visual inspection, all signals were normalized to z-scores (i.e., the mean was subtracted and then divided by the standard deviation). (Right) The fNIRS signals after motion correction. The slow drift motions (e.g., the red square box) and spikes (e.g., the red ellipse) were corrected.

**Figure S2**. Behavioral performance of the landmark task against its control in terms of reaction time and accuracy. Participants showed less accuracy and slower response time in landmark task.

**Figure S3**. The thresholded activation t-maps of the landmark control condition.

**Figure S4.** The thresholded activation t-maps of the picture-naming and landmark tasks in two subsets. Similar activation patterns were observed.

**Figure S5.** The hemispheric difference in the averaged hemodynamic responses within the ROIs under the landmark control

Condition

**Figure S6.** Hemispheric differences in HbR within ROIs. A) The left ROI showed a greater decrease than the right ROI in the picture naming task (t = -2.59 p<0.05). B) The right ROI showed a greater decrease than the left ROI in the landmark condition in the landmark task (t = 3.79, p<0.001). The red and black rectangle represents the duration of the instruction and task periods, respectively. The error bar represents a 95% confidence interval.

**Figure S7.** The relationship between the picture-naming and landmark task in terms of the degree of laterality index derived from HbR. Dissociation of the laterality index between the picture-naming task and the landmark task.

.


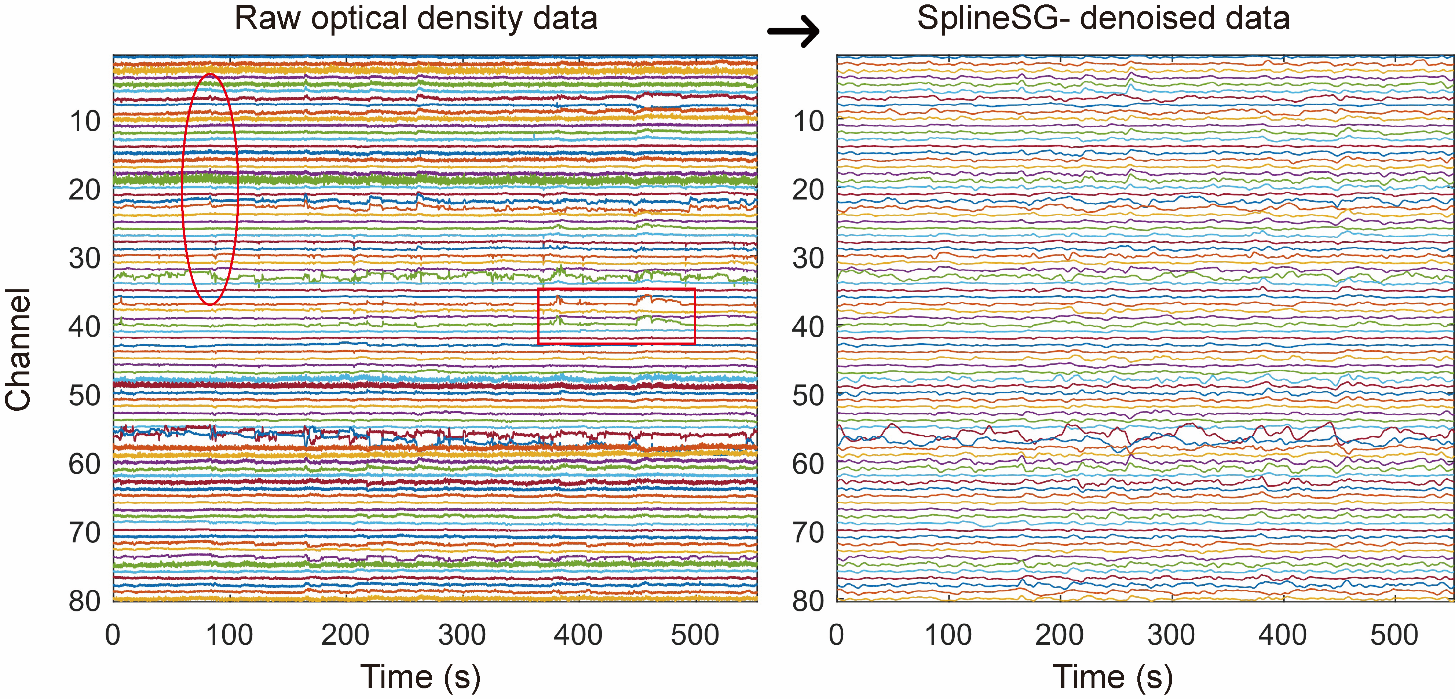


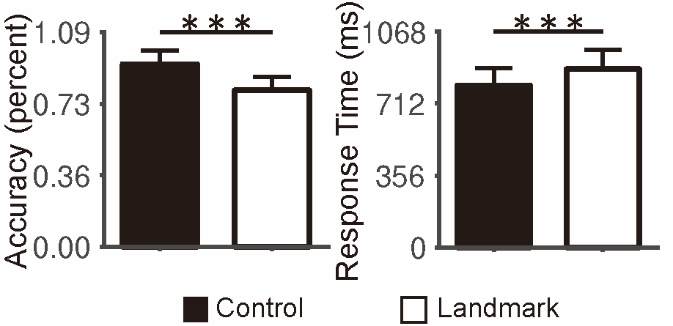


*
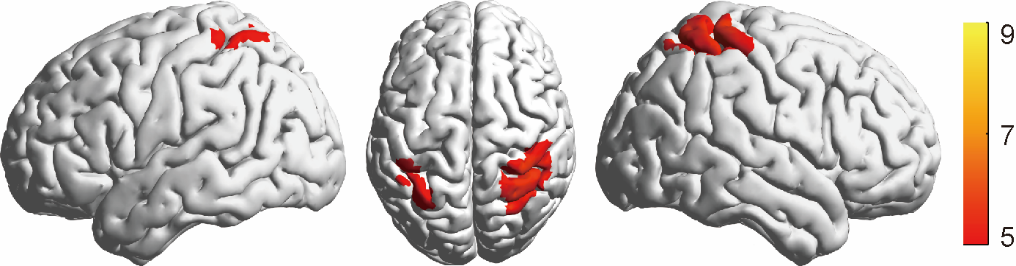
*


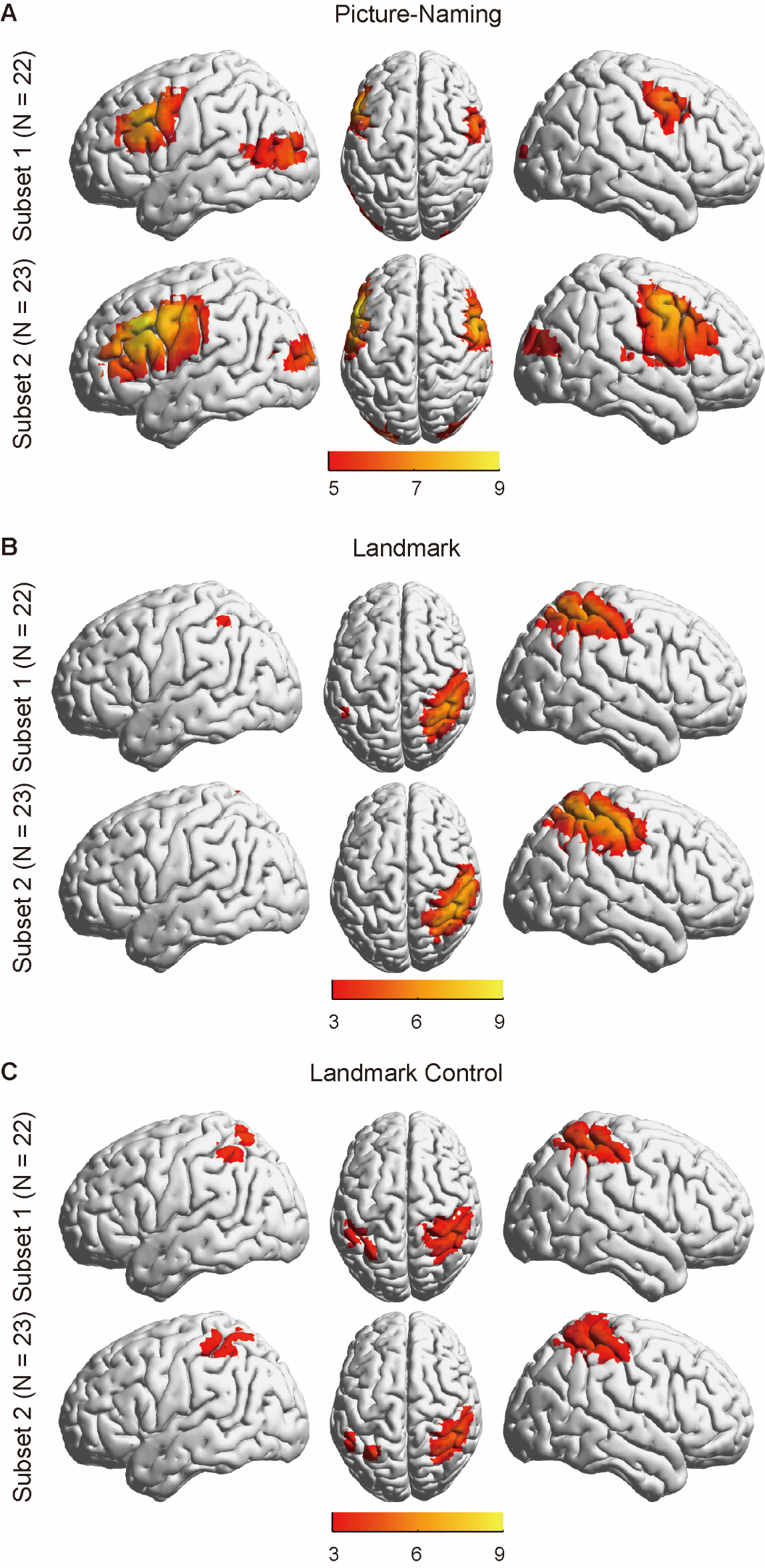


*
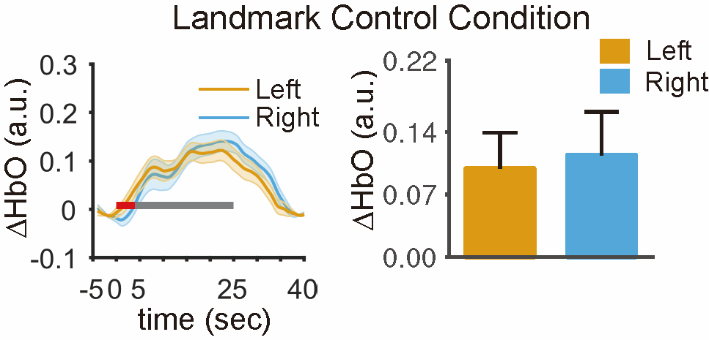
*


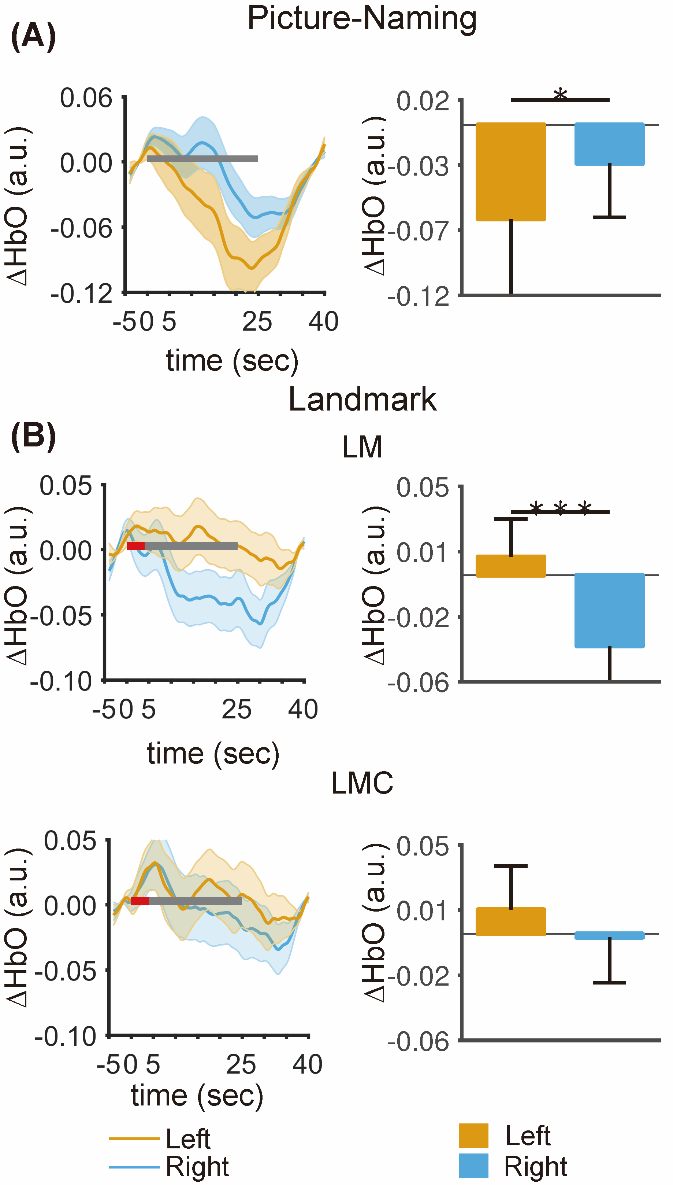


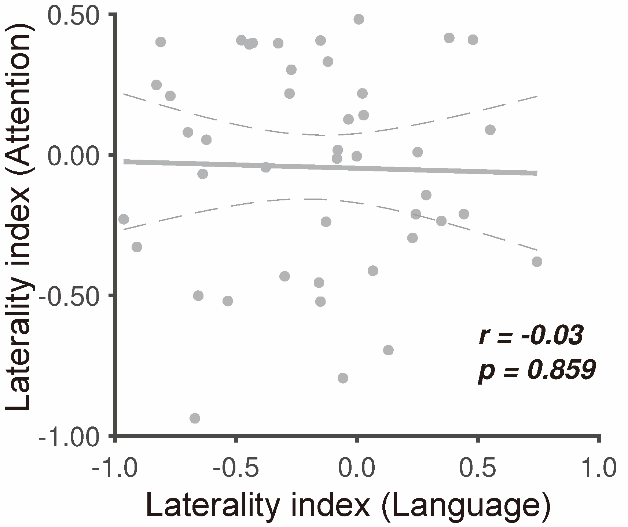

Supplement: Supplementary file 1 [file Data_Sheet_1.docx]
