# Supplementary figures and images for: Hemispheric Lateralization of Visuospatial Attention Is Independent of Language Production on Right-Handers: Evidence From Functional Near-Infrared Spectroscopy
Source: Front Neurol. 2022 Jan 14;12:784821. doi: 10.3389/fneur.2021.784821 (PMC8795708; doi:10.3389/fneur.2021.784821)

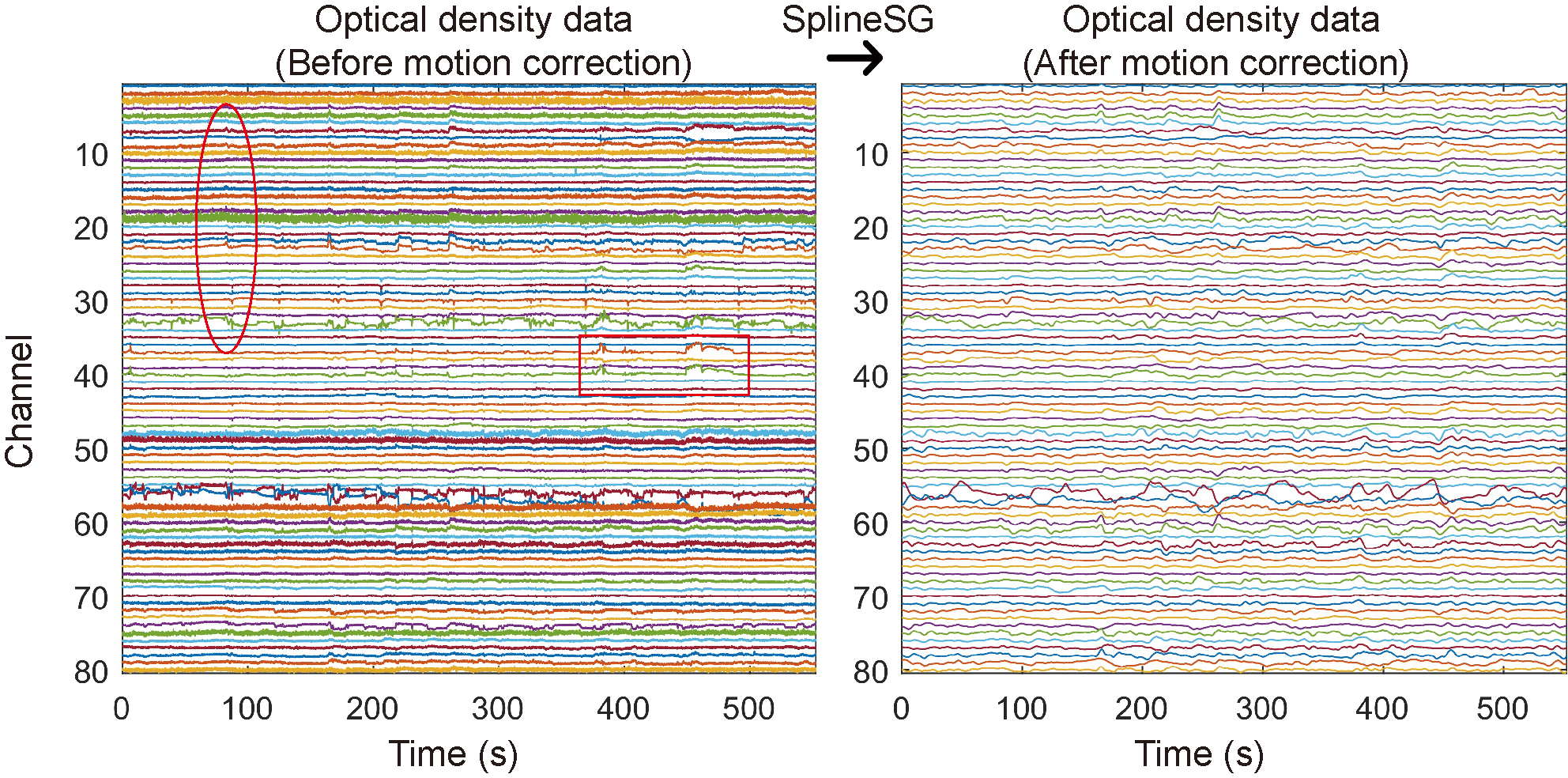

Supplement: Supplementary file 2 [file Image_1.TIF]

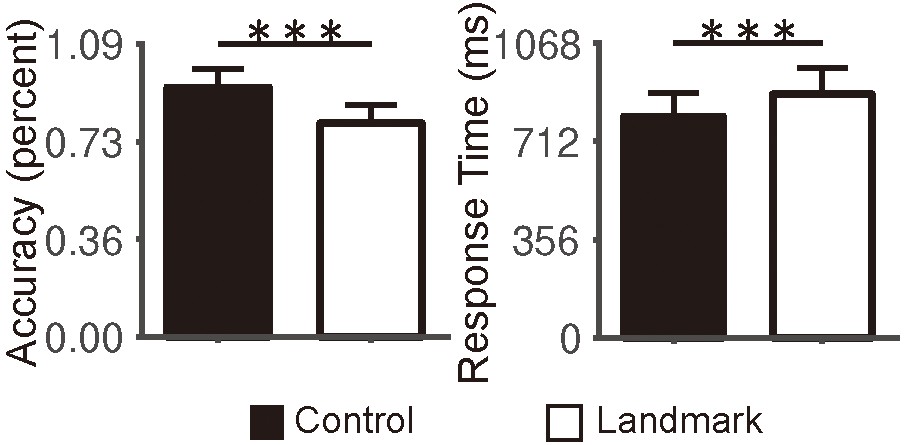

Supplement: Supplementary file 3 [file Image_2.TIF]

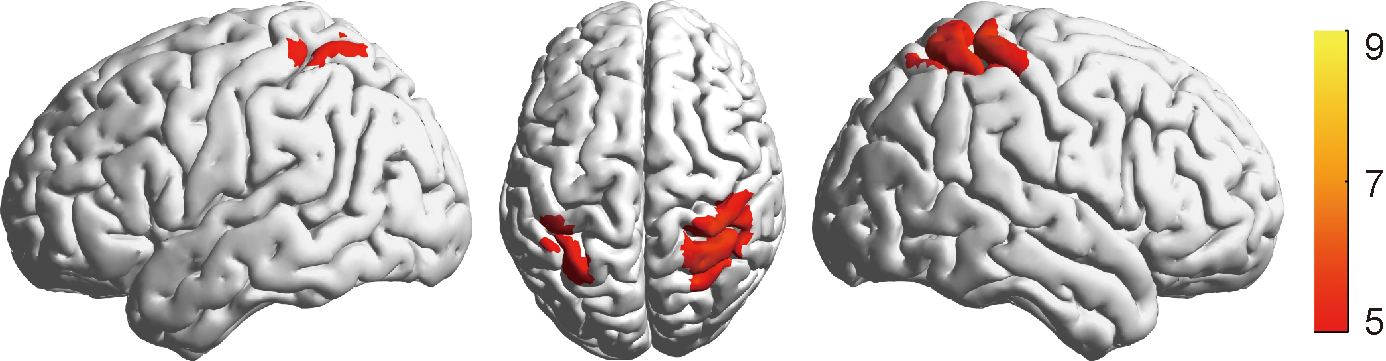

Supplement: Supplementary file 4 [file Image_3.TIF]

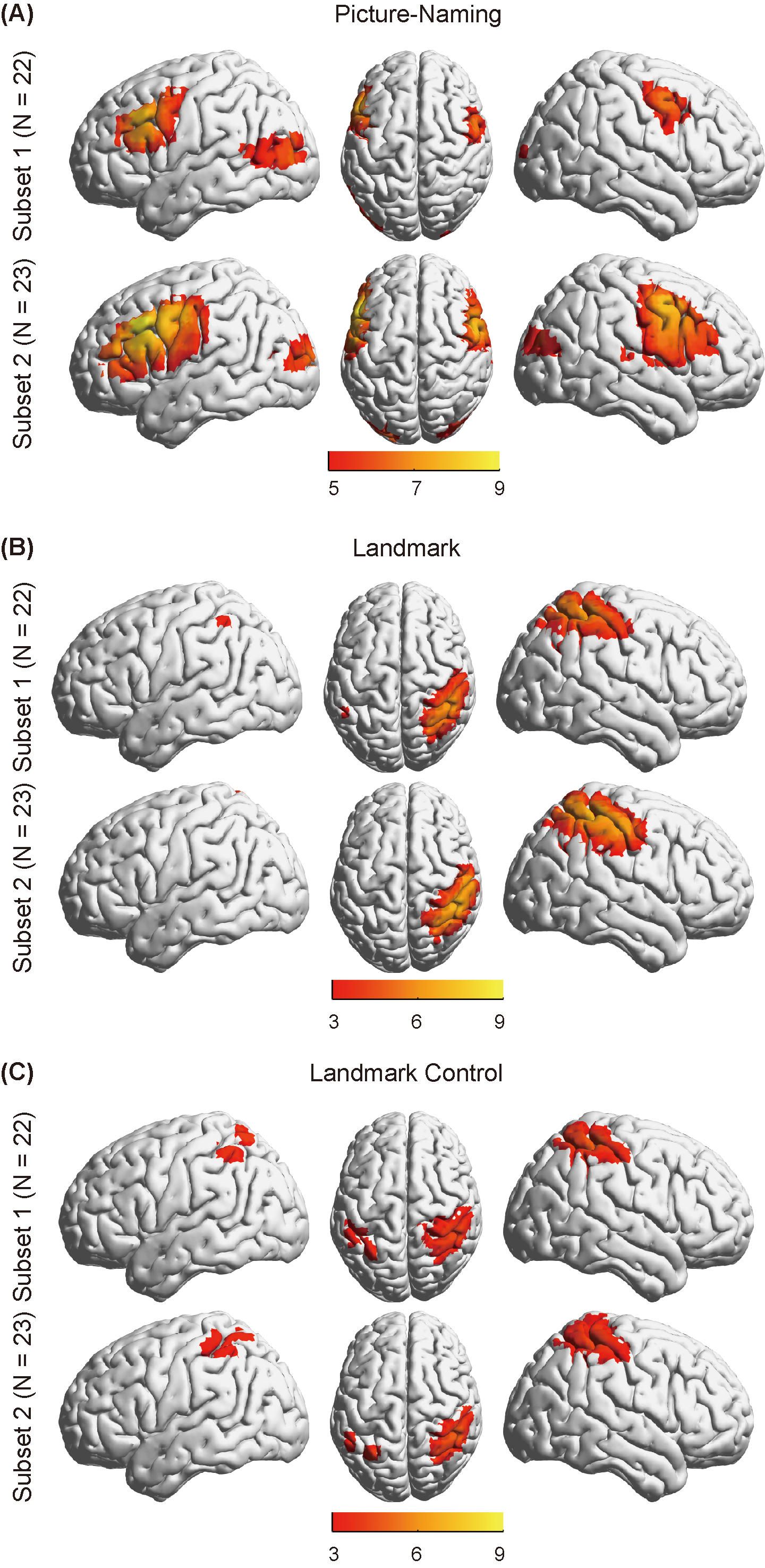

Supplement: Supplementary file 5 [file Image_4.TIF]

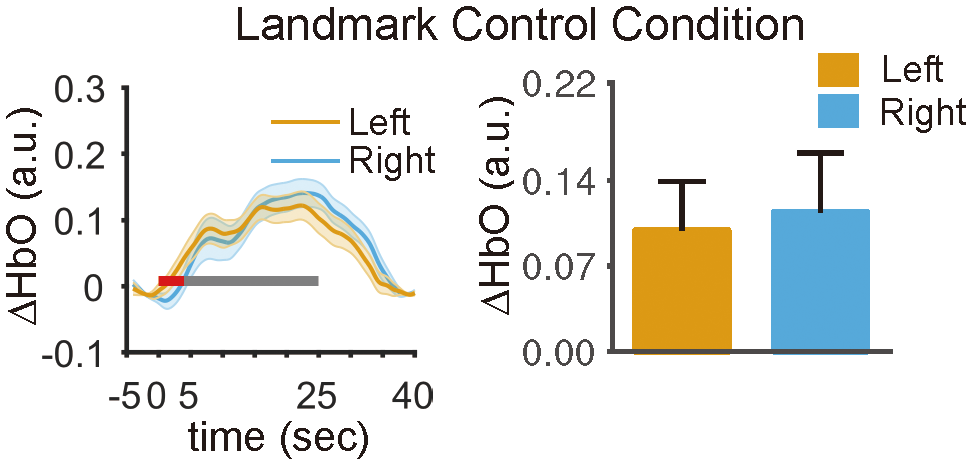

Supplement: Supplementary file 6 [file Image_5.TIF]

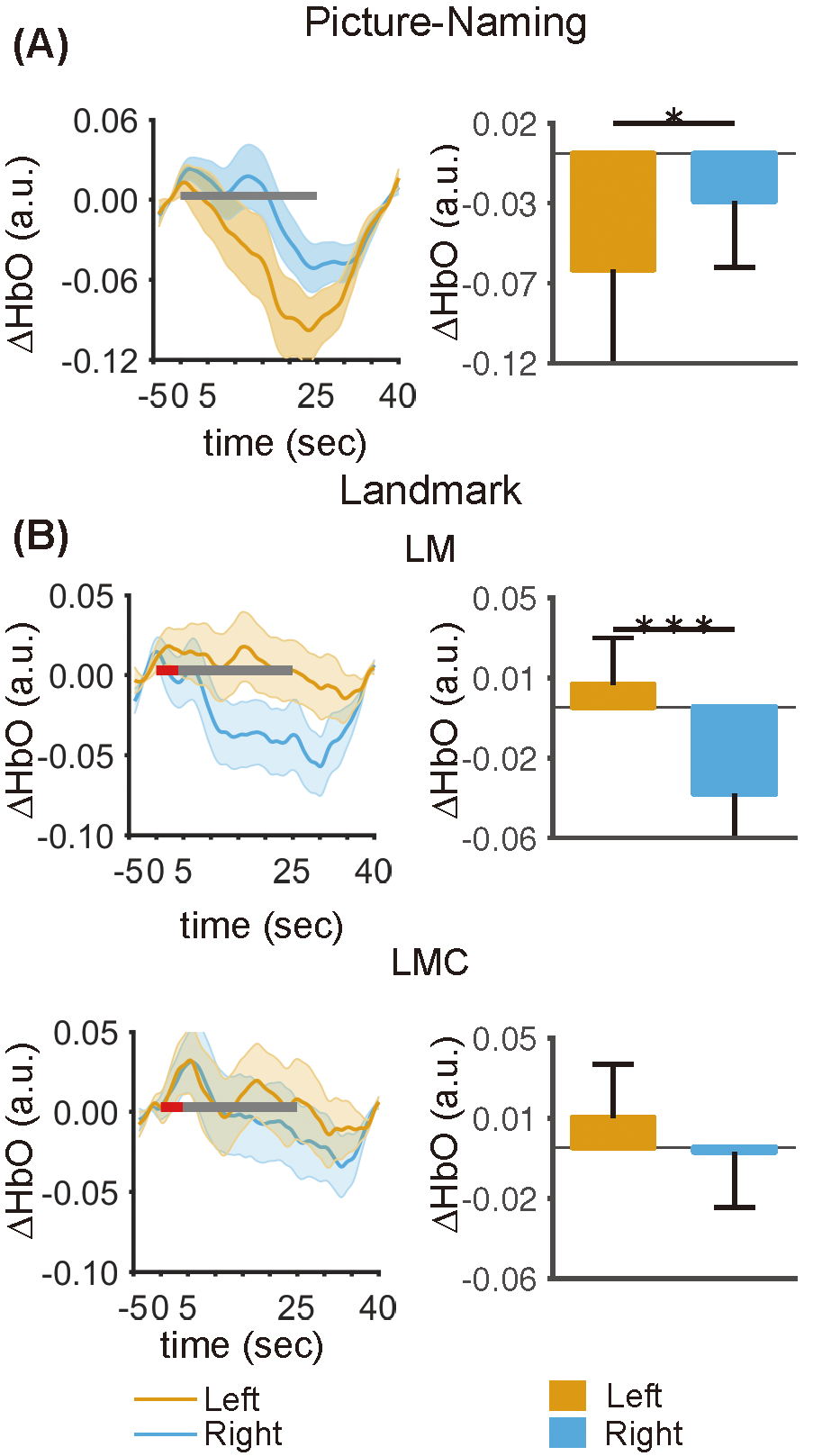

Supplement: Supplementary file 7 [file Image_6.TIF]

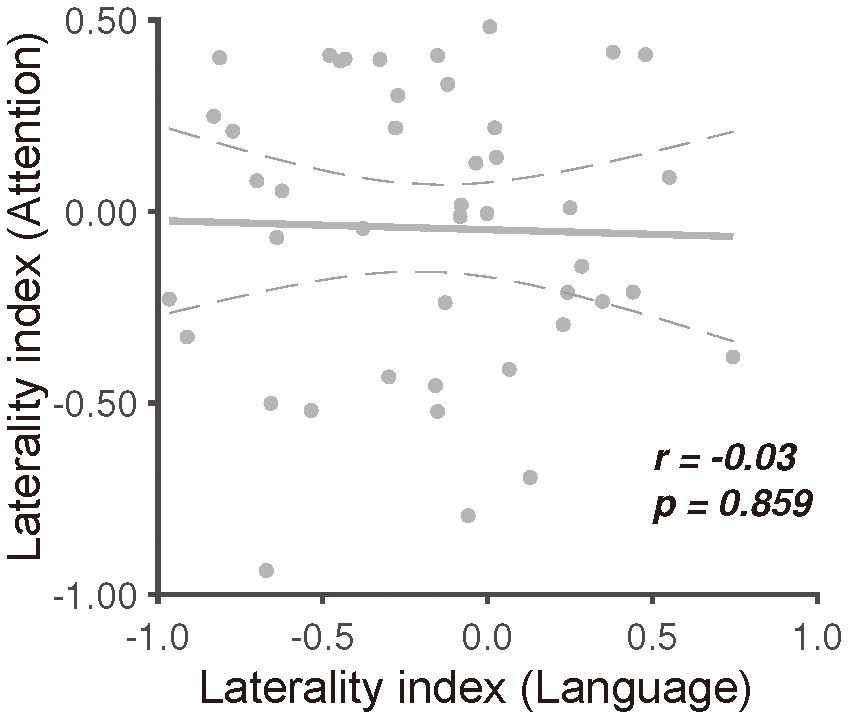

Supplement: Supplementary file 8 [file Image_7.TIF]
